# Symptomatic May-Thurner Syndrome Without Deep Venous Thrombosis

**DOI:** 10.7759/cureus.6178

**Published:** 2019-11-18

**Authors:** Keegan S McNally, Latha Ganti, Vanessa I Diaz, Amanda L Webb, George Alvarez

**Affiliations:** 1 Emergency Medicine, University of Central Florida College of Medicine / Hospital Corporation of America Graduate Medical Education Consortium of Greater Orlando, Orlando, USA; 2 Emergency Medicine, Envision Physician Services, Orlando, USA; 3 Emergency Medicine, University of Central Florida / Osceola Regional Medical Center, Orlando, USA; 4 Internal Medicine, University of Central Florida College of Medicine, Windermere, USA

**Keywords:** may-thurner syndrome

## Abstract

The authors present a case of symptomatic May-Thurner syndrome in the absence of a deep venous thrombosis. This is an unusual case, as most cases are diagnosed with a deep venous thrombosis as the underlying finding. The clinical presentation and suggested diagnostic workup are discussed. A key point is the need to consider this frequently under-diagnosed condition. Optimal management is often with a stent, but if not diagnosed, the patient can develop unnecessary clot burden, be placed on lifelong anticoagulation, or both.

## Introduction

May-Thurner syndrome is characterized by the right common iliac artery crossing over the left iliac vein and compressing it against the lumbar vertebral body, which results in the progressive fibrosis of the left iliac vein, leading to occlusive symptoms [1]. Most commonly, the patient will present with symptoms of an underlying deep venous thrombosis, including pain, swelling, and discoloration of the extremity [2]. May-Thurner Syndrome is proposed to be the reason why deep venous thrombosis is five times more likely in the left lower extremity versus the right lower extremity [3]. Patients may present in a spectrum of disease, including asymptomatic extending to extensive venous thrombus in the left lower extremity and/or pulmonary embolus [4].

## Case presentation

**Figure 1:**
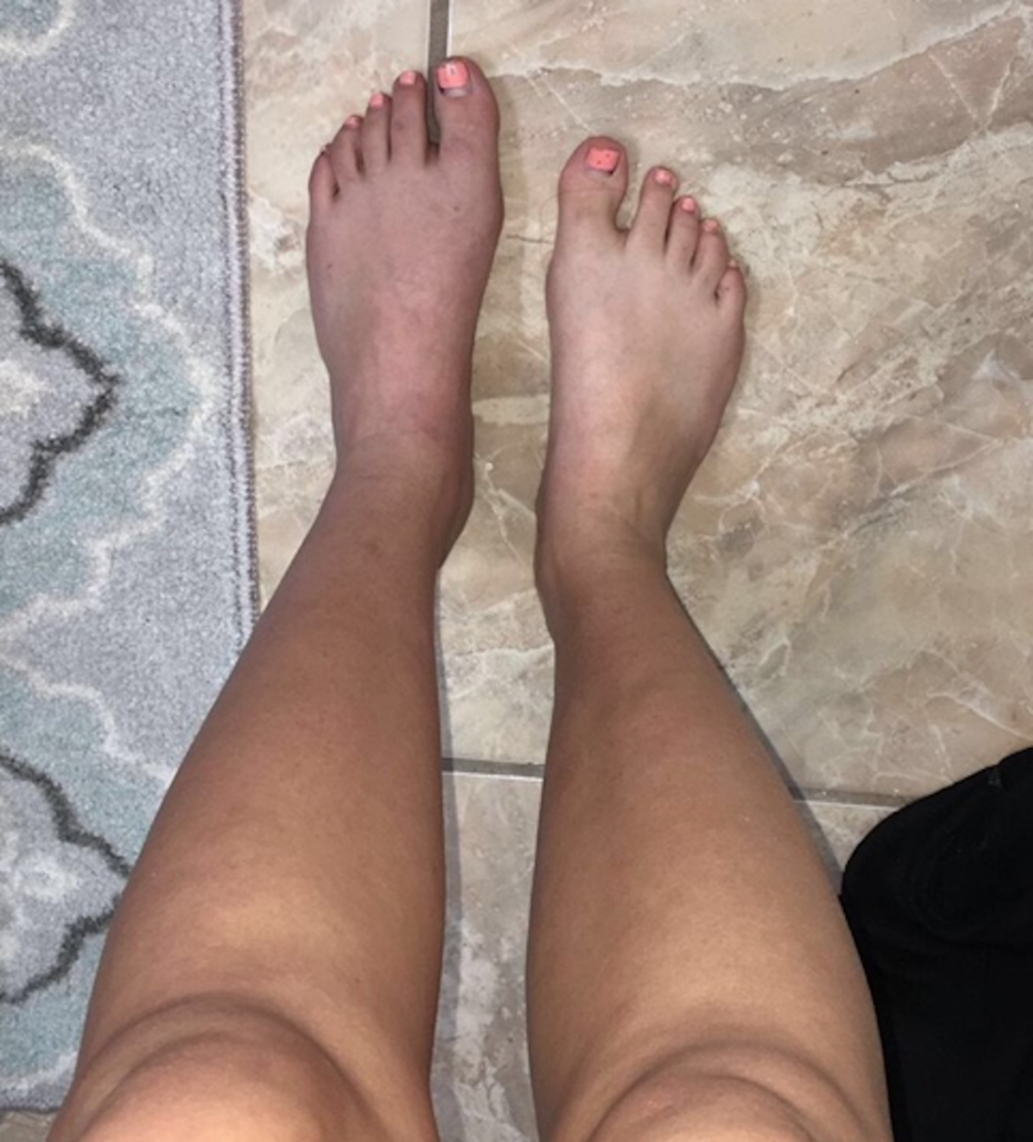
Picture taken at home by patient demonstrating cyanosis of the left lower extremity, most noticeable on the dorsum of the foot.

An 18-year-old female patient with no past medical, surgical, or obstetric history, on no medications, presented to the emergency department (ED) with a chief complaint of left lower extremity pain. The patient described a dull, throbbing sensation that occurred intermittently, lasting from 30 minutes to 60 minutes, first starting one month prior and worsening over that time. The pain was localized to the dorsum of the foot, ankle, and anterior shin. It was associated with an intermittent bluish discoloration of the extremity; however, this was not present at the time of presentation. The patient shared a photo of the discoloration she had noted previously (Figure 1).

The patient had been seen three times for this pain in the past month. The first presentation was at an urgent care center, where she was discharged with ibuprofen and follow-up with a primary care physician in the area, with instructions to present to an ED if symptoms worsened. When symptoms worsened, the patient presented to a pediatric ED, where she underwent an X-ray of her left foot and ankle with no abnormalities, and provided orthopedic specialty follow-up. At the orthopedic clinic, the patient was provided with exercise instructions for ankle sprains, with instructions that further testing might be required if the pain did not improve. The patient presented to the ED after episodes of pain, discoloration, and change in temperature became more frequent and began to limit her ability to exercise in school, and the patient started to require intermittent use of crutches.

On examination, the patient’s left lower extremity was atraumatic, of normal color, and without pain to palpation over bony prominences or when the ankle joint was manipulated. Pulses were palpable over the dorsalis pedis and posterior tibialis arteries. No swelling was noted. The patient’s left lower extremity appeared colder to the touch than the right lower extremity, and the patient had pain when asked to walk without crutches and did so with an antalgic gait. Reflexes, strength, and sensation were all intact in both lower extremities. The remainder of the patient’s physical exam revealed no abnormalities, including neurologic, cardiovascular, abdominal, and respiratory exams. A complete blood count, basic metabolic panel, and urinalysis were within normal limits, and beta-human chorionic gonadotropin (beta-hCG) was negative.

**Figure 2:**
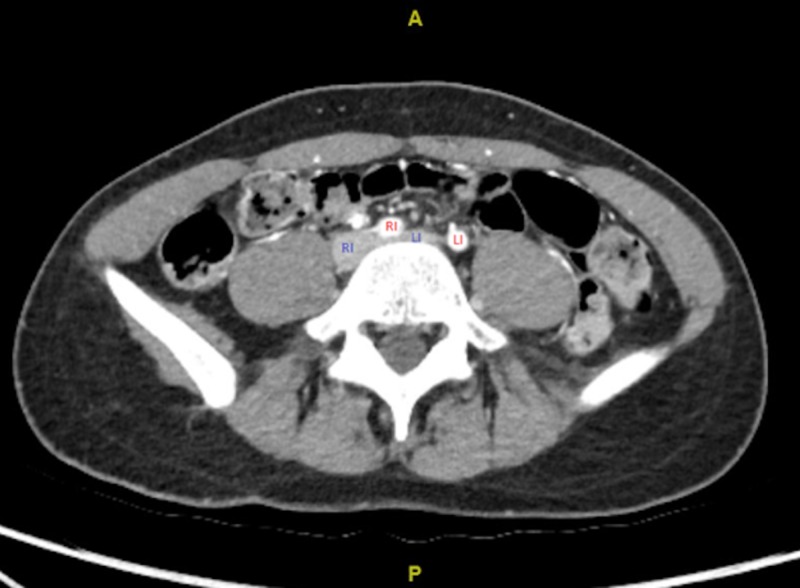
CTA aorta with runoff to lower extremity noting flattening of the left common iliac vein (blue LI) by the right iliac artery (red RI). Right common iliac vein (blue RI) and left iliac artery (red LI) also noted.

After the exam and laboratory work were found unremarkable, the patient was sent for lower extremity venous and arterial doppler. There was no evidence of deep venous thrombosis (DVT) in the left lower extremity common femoral, femoral, popliteal, posterior tibial, or proximal greater saphenous veins. Arterial doppler revealed, “no plaque is noted neither is thrombus. Flow appears to be within normal limits. However, velocities may be somewhat decreased below the knee on the left side”. This study, although within normal limits, prompted computed tomography angiography (CTA) of bilateral lower extremity for further evaluation and correlation. CTA revealed “mild stenosis of the left common iliac vein, secondary to compression from the overlying right common iliac artery. This finding may be seen in the clinical setting of May-Thurner syndrome; however, there were no obvious iliac venous DVT and no pelvic venous collaterals.” This finding is shown below (Figure 2).

**Figure 3:**
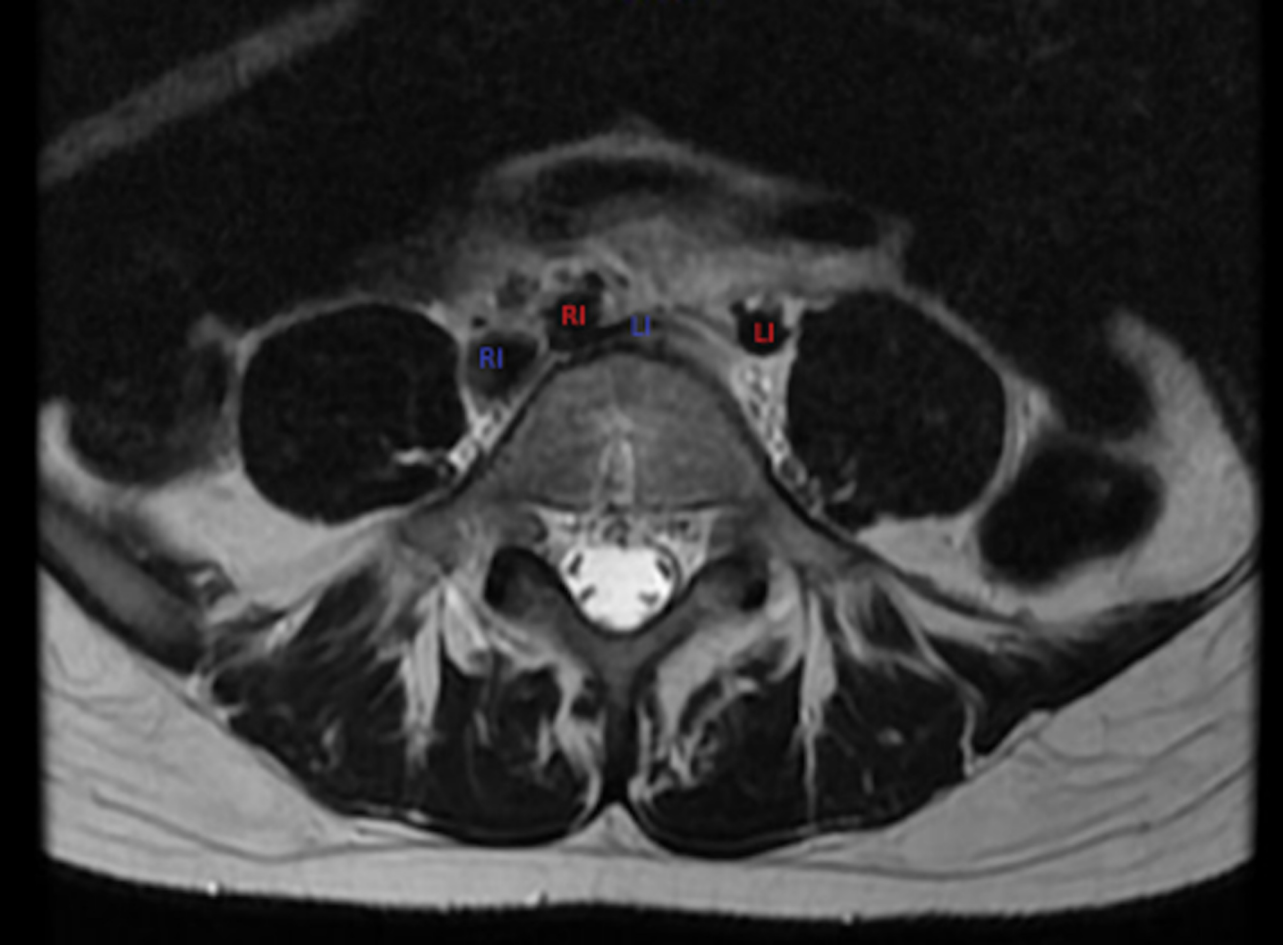
MRI of the lumbar spine again noting flattening of the left common iliac vein (blue LI) by the right iliac artery (red RI). Right common iliac vein (blue RI) and left iliac artery (red LI) also noted.

Vascular surgery was called and notified about the case from the emergency department. The patient was admitted to the internal medicine service with a vascular surgery consult from the emergency department in stable condition, with intact pulses in bilateral lower extremities. The patient received an MRI of the lumbar spine without contrast the next day to further evaluate the area of concern and to rule out other causes for the patient’s symptoms. This study again revealed flattening of the left iliac vein by the right iliac artery. This finding is shown below (Figure 3):

The patient was taken for angioplasty of the left common iliac vein the next day, and an 80% stenosis of the vein was discovered during the procedure by venogram. After angioplasty, repeat venogram intraoperatively showed minimal recoiling of 50%. The patient tolerated the procedure well and noted the next morning, no pain or cyanosis in her left lower extremity while standing in the shower, which was a very common time for her to have the pain prior to surgery. The patient was discharged the same day with vascular surgery followup in two weeks. The patient was not started on anticoagulation, as no evidence of actual thrombus formation was seen during her stay. The patient was asymptomatic on the one-month followup, with no episodes of cyanosis or deep venous thrombosis.

## Discussion

May-Thurner syndrome (MTS) is a common but rarely diagnosed disorder involving left common iliac vein compression by the right iliac artery, with a suspected prevalence of 24% in the general population in retrospective studies of computed tomography scans, and 22% in cadaveric studies [2,3]. This occurrence seemingly accounts for the increased rate of deep venous thrombosis in the left lower extremity, regardless of weight, age, gender, or other identifiable factors [4]. Although the prevalence of left iliac vein compression appears to be common, a combination of anchoring bias and resource management may predispose the disorder to a lower diagnosis rate. A patient may present with the signs and symptoms of a DVT, and be diagnosed with one through various methods, most commonly a lower extremity ultrasound of the deep veins, but the workup may stop at this point. MTS requires expensive testing, including magnetic resonance imaging (MRI) or computed tomography (CT) or both as seen above, which carry both financial and radiation burden to a patient. A patient may never be diagnosed with MTS after this workup, as clinicians may stop further imaging for cost and resource purposes. This, however, may be short-sighted, as MTS has been theorized to be a reason for recurrent left-sided DVT after anticoagulation [5]. Stent placement has been shown to reduce the risk of recurrent DVT in patients with MTS, even if continuous anticoagulation is given [6]. If the rate of MTS is so prevalent in radiographic and cadaveric studies, it may be cost-efficient in the long-term to screen patients with left-sided DVT of the lower extremity with pelvic MRI or CTA to assess the need for stent placement. Reducing future clot-burden, or possible need for life-long anticoagulation may outweigh the initial investment cost; however, a thorough financial analysis is needed.

## Conclusions

A high index of suspicion is needed to diagnose May-Thurner syndrome. This diagnosis should be considered in anyone who presents with left lower extremity occlusive symptoms to prevent undue morbidity. 
